# Degradation of Folic Acid in the Composition of a Conjugate with Polyvinylpyrrolidone and Fullerene C_60_ Under UV and E-Beam Irradiation

**DOI:** 10.3390/molecules30132718

**Published:** 2025-06-24

**Authors:** Alina A. Borisenkova, Dmitriy V. Baykov, Anna V. Titova, Vadim V. Bakhmetyev, Maria A. Markova, Zhanna B. Lyutova, Anton V. Popugaev, Vladislav S. Khaleev, Victor P. Sedov

**Affiliations:** 1 Radiation Technology Department, St. Petersburg State Institute of Technology (Technical University), 190013 St. Petersburg, Russia; 2 Petersburg Nuclear Physics Institute named by B.P. Konstantinov of National Research Centre “Kurchatov Institute”, 188300 Gatchina, Russia; 3 Department of Theory of Materials Science, St. Petersburg State Institute of Technology (Technical University), 190013 St. Petersburg, Russia; 4 Infochemistry Scientific Center, ITMO University, 191002 St. Petersburg, Russia

**Keywords:** folic acid, fullerene, UV irradiation, photodegradation, radiolysis, radiation chemical yield

## Abstract

Folic acid (FA) is used as a targeting ligand for targeted drug delivery to tumor cells, some types of which overexpress folate receptors on their surface. However, while the preparation of conjugates containing FA may comprise a multi-step process, FA presents low photostability under UV irradiation. In addition, FA undergoes radiolysis under the action of ionizing radiation, which is utilized for drug sterilization. In this study, we investigate the stability of FA in a conjugate (FA-PVP-C_60_) with fullerene C_60_ and polyvinylpyrrolidone under the action of UV (205–400 nm) and electron irradiation (doses from 2 to 8 kGy) at different pH (4.5, 7.2, 10.7). The degradation of FA is studied using fluorescence and UV–Vis spectroscopy. It is found that the fullerene C_60_ in the FA-PVP-C_60_ conjugate suppresses the degradation of FA during both photolysis and radiolysis, which is confirmed by the decrease in the quantum yield of fluorescence and the radiation chemical yield of FA destruction accompanied by increasing fullerene content in the conjugate (from 2.8 to 10 wt.%).

## 1. Introduction

The use of targeting vectors for targeted drug delivery helps to reduce the minimum effective dose and associated drug toxicity while also increasing therapeutic efficacy [1]. The cellular uptake of folic acid (FA) is mediated by folate receptors, whose amount is elevated in some types of cancer cells [2,3]. Therefore, FA is considered a promising targeting agent in the composition of targeted drug delivery systems for the diagnosis and therapy of certain tumors [4,5,6,7,8,9,10,11,12,13]. We have previously shown that a conjugate of FA with fullerene C_60_ and polyvinylpyrrolidone (FA-PVP-C_60_) exhibited greater accumulation in tumor cells with increased expression of folate receptors [14]. However, FA is sensitive to the effects of ultraviolet (UV) light [15,16,17,18,19] and ionizing radiation [20,21,22,23], and the exposure of human blood to ultraviolet A (UVA, 320–400 nm long-wave range) radiation has been shown to result in the photodegradation of folate both in vitro and in vivo [24,25]. At the same time, in order to preserve the targeting properties of FA associated with its absorption by cells through receptor-mediated endocytosis, ensuring the integrity of the molecular structure of FA is important [26].

**Figure 1:**
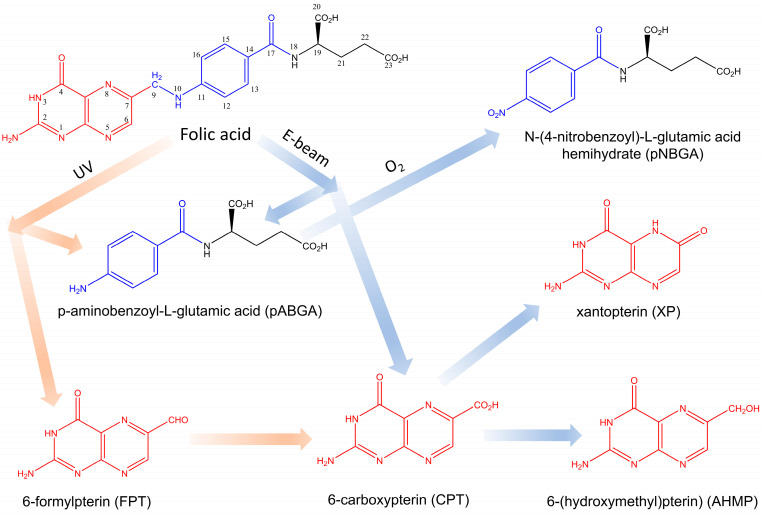
UV (orange arrows) and radio (blue arrows) FA degradation pathways.

The 9-10 bond between methylpterin (MPT) fragments and p-aminobenzoyl-L-glutamic acid (pABGA) in FA is most sensitive to UV and ionizing radiation (Figure 1) [20,21,27,28,29,30]. According to literature data, the first stage of photodegradation involves photooxidation of FA, initiated by excited states of the acid itself, leading to the formation of pABGA and 6-formylpterin (FPT) [15]. In the second stage, the presence of FPT sensitizes the FA, which leads to the acceleration of its degradation. At the third stage of photodegradation of FA, oxidation of FPT to 6-carboxypterin (CPT) occurs.

In contrast to the processes of FA degradation under the influence of UV radiation, the process of FA radiolysis has been studied to a lesser extent. As reported by Araujo et al. [21], during radiolysis, the FA molecule degrades to form pABGA and CPT, bypassing the stage of FPT accumulation (Figure 1). Additionally, radiolysis products such as xanthopterin [21], as well as 6-(hydroxymethyl)pterin (AHMP) [20] and the oxidation product of pABGA—N-(4-nitrobenzoyl)-L-glutamic acid (pNBGA)—were detected.

At the same time, the products of the radiolysis and photolysis of FA have a photosensitizing effect on FA, thus accelerating the process of decomposition [18]. In addition, the photolysis of FA is dependent on the nature of the solvent [31] and on the pH of the environment, and it has been found that the rate of photolysis gradually decreases when moving from an acidic to an alkaline environment [28].

Radiodegradation processes may limit the use of FA as a targeting ligand in drugs [32] with radioactive isotopes, as well as in drugs subjected to radiation sterilization. Photodegradation of FA may complicate the synthesis of conjugates based on it and require special storage conditions. Notably, fullerene C_60_—which is known for its antioxidant properties in the dark—can improve the photo- and radiostability of FA in the FA-PVP-C_60_ conjugate. The main advantages of using fullerene as an antioxidant include the fact that it can react with many radicals without being consumed and is also capable of localizing inside the cell in the mitochondria and other places in the cellular compartments where free radicals are produced in disease states [32]. It has also been shown that water-soluble fullerene derivatives act as radioprotectors due to their antioxidant properties. [33,34].

In this line, it has been shown that the photostability of FA can be improved through the presence of antioxidants [35,36,37,38], surface-active compounds [39], and by binding to proteins [25,36,40,41,42] or forming complexes with biopolymers [43] in the composition of various preparations. On the other hand, it is known that the photoexcitation of fullerene by light in a wide range of wavelengths can generate active forms of oxygen, which is a property leveraged to create fullerene-containing drugs for photodynamic therapy [44,45,46]. In particular, singlet oxygen, which is produced after the excitation of the photosensitizer, can destroy FA [16].

Despite the numerous ongoing research endeavors regarding the use of FA as a targeting ligand, there is a notable scarcity of attention from researchers about the stability of FA in targeted drugs during synthesis, application, and storage. The aim of the present work was to investigate the effect of the content of fullerene C_60_ (from 2.8 to 10 wt.%) on the photo- and radiostability of FA in the FA-PVP-C_60_ conjugates at different pH (from 4.5 to 10.7).

## 2. Results and Discussion

**Figure 2:**
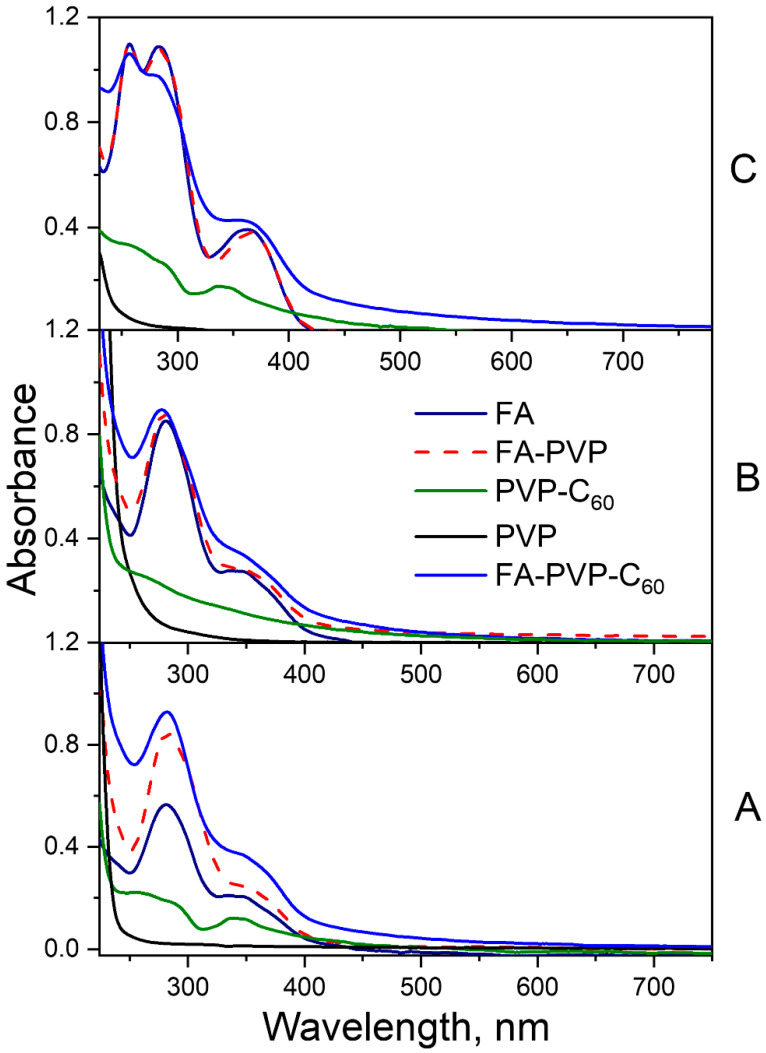
Absorption spectra of the FA-PVP-C_60_ conjugate, intermediate synthesis products FA-PVP, PVP-C_60_, PVP, and FA at pH 4.5 (**A**), pH 7.2 (**B**) and pH 10.7 (**C**).

The absorption spectrum of FA at pH 4.5 and 7.2 presented two maxima in the ultraviolet B (UVB, 280–320 nm medium-wave range) and UVA spectral regions (Figure 2A,B), and therefore this molecule can be excited by solar irradiation. The absorption bands at 280 and 350 nm are related to the π–π* and n–π* electron transitions in pterin (PT) and pABGA fragments, respectively [18]. Unlike the acidic form, where the high-energy band of PT and pABGA overlap, the basic form of FA had three absorption bands at 256 nm, 283 nm and 365 nm (Figure 2C) [47]. The absorption spectra of FA-PVP-C_60_ does not differ significantly from that of native FA [19]. The UV–Vis spectra in DMF of both the intermediate PVP-C_60_ conjugates and the FA-PVP-C_60_ conjugates with different fullerene contents (Figure S1, Supplementary Materials) showed a maximum at about 330 nm. The absence of a wavelength shift in the characteristic C_60_ absorption maximum in DMF suggests the formation of a non-chemical bond between the fullerene and the conjugate components [48]. In the FTIR spectra of FA-PVP-C_60_ conjugates with different fullerene contents (Figure S2, Supplementary Materials), the bands characteristic of C_60_ [49] are masked by the bands of bond vibrations of the PVP. In all FA-PVP-C_60_ conjugates FTIR spectra, a slight red shift in the strong absorption peak at 1653 cm^−1^, responsible for the C=O stretching vibration in PVP, was observed [50]. The magnitude of this shift was small and did not correlate with the fullerene content in the conjugate, indicating the absence of covalent bond formation between fullerene and PVP. Thus, the conjugation of the components of the conjugate with PVP occurred through the formation of a charge transfer complex involving the C=O bond of the pyrrolidone ring of PVP [51]. The XRD spectra of all conjugates contained only two broad peaks characteristic of pure PVP (Figure S3, Supplementary Materials) [52]. In this case, we did not observe the diffraction maxima characteristic of fullerene C_60_ [53] and FA [54]. Also, a slight change in the interplanar distances of the conjugates was observed compared to pure PVP.

### 2.1. Photodegradation of FA in FA-PVP-C_60_

**Figure 3:**
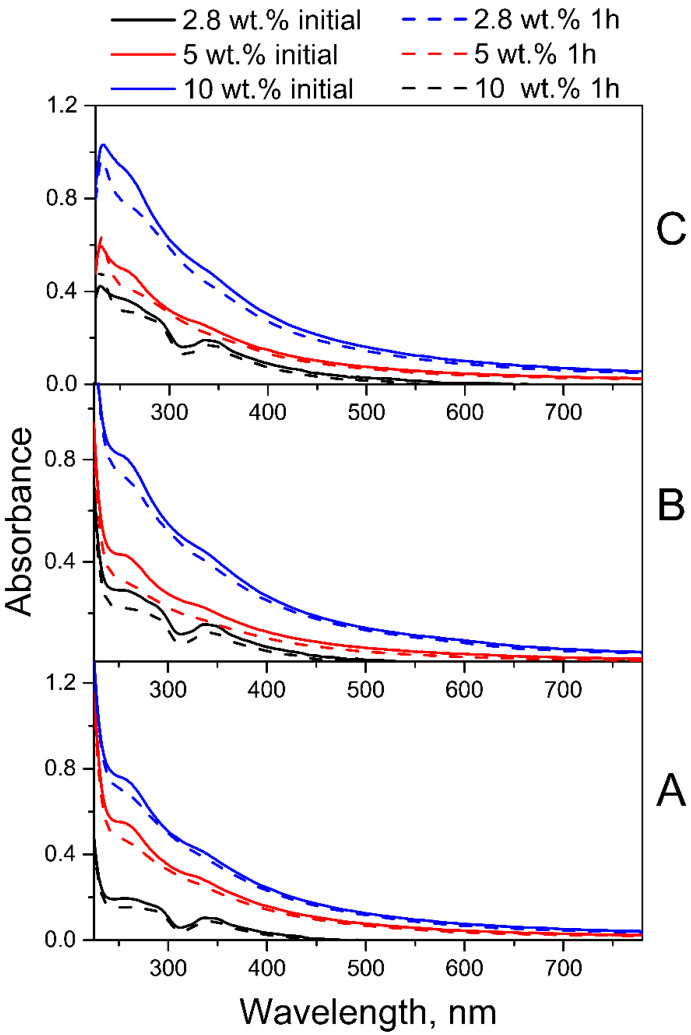
Absorption spectra of C_60_-PVP conjugates with fullerene content of 2.8, 5 or 10 wt.% before and after 1 h of UV irradiation at pH 4.5 (**A**), pH 7.2 (**B**) and pH 10.7 (**C**).

As FA has absorption maxima in both UVA and UVB spectral regions, two lamps with ranges of 300–400 nm (λ_max_ = 350 nm) and 205–315 nm (λ_max_ = 254 nm) were used simultaneously for photolysis. The photostability of fullerene in intermediate PVP-C_60_ conjugates was preliminarily estimated under UV irradiation conditions in the same spectral range for 1 h. The absorption spectra (Figure 3) before and after 1 h of UV irradiation confirmed the photostability of fullerene C_60_ in the intermediate PVP-C_60_ conjugates with fullerene content from 2.8 to 10 wt.% at different pH of aqueous solutions.

#### 2.1.1. Neutral Aqueous Solutions

**Figure 4:**
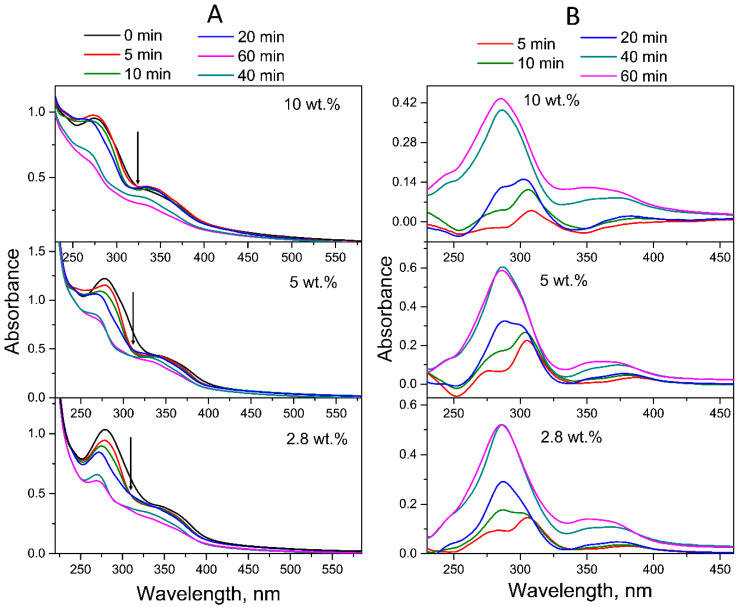
(**A**) Time evolution of the absorption spectra of the FA-PVP-C_60_ conjugates in air-equilibrated aqueous solutions (pH 7.2) under UV irradiation (240–400 nm). Arrows indicate the isosbestic points. (**B**) Experimental difference (ED) spectra obtained by subtracting the spectrum after 5–60 min of photolysis from the FA-PVP-C_60_ conjugates initial spectrum.

Figure 4 shows the time evolution of the UV–Vis absorption spectra of FA-PVP-C_60_ conjugates with different fullerene contents in neutral aqueous solutions (pH 7.2). As can be seen from Figure 4A, with increasing UV irradiation time, the absorption of the band at 280 nm gradually decreased, while the absorption of the band at 350 nm gradually increased. This behavior causes the appearance of an isosbestic point at 311 nm, which indicates the formation of FA photodegradation products [55]. Upon UV irradiation, in addition to a decrease in absorption, the absorption maxima of FA underwent a blue shift: from 284 and 350 nm, the bands shifted to 272 and 342 nm, respectively. Moreover, the decrease in absorption was more pronounced for the band at 284 nm, corresponding to the pABGA fragment. It is known that FA absorbs approximately 3.8 times stronger at 280 nm than at 350 nm [27].

According to the literature data, the first stage of photodegradation involves the photooxidation of FA, initiated by excited states of the acid itself, leading to the formation of pABGA and FPT [15]. During the second stage, the presence of FPT sensitizes the FA, which leads to the acceleration of its degradation.

To qualitatively represent the influence of fullerene C_60_ on the process of photodegradation of FA in the conjugates, experimental difference (ED) spectra were obtained by subtracting the spectra of conjugates irradiated for 5–60 min from their native spectra. As can be seen in Figure 4B, the ED spectrum of the all-FA-PVP-C_60_ conjugates neutral solution irradiated for 5 min contains maxima at 278, 310 and 365 nm, characteristic of FPT [56]. Then, after 20 min of irradiation, the ED spectrum of the conjugate with 2.8 wt.% fullerene contained maxima at 290 and 350 nm, characteristic of CPT [57], which has a lower quantum yield (QY) of photodegradation compared to FA and FPT. Thus, the third stage of FA photodegradation, oxidation of FPT to CPT, began after 10 min of irradiation of the FA-PVP-C_60_ conjugate containing 2.8 wt.% of fullerene. Also, the ED spectra of FA-PVP-C_60_ conjugates (2.8 wt.%) irradiated for 40 and 60 min were virtually identical, indicating that by 40 min of irradiation, virtually all FA had degraded. With increasing concentration of the fullerene in the conjugate, it can be noted that the FPT oxidation stage began later.

**Figure 5:**
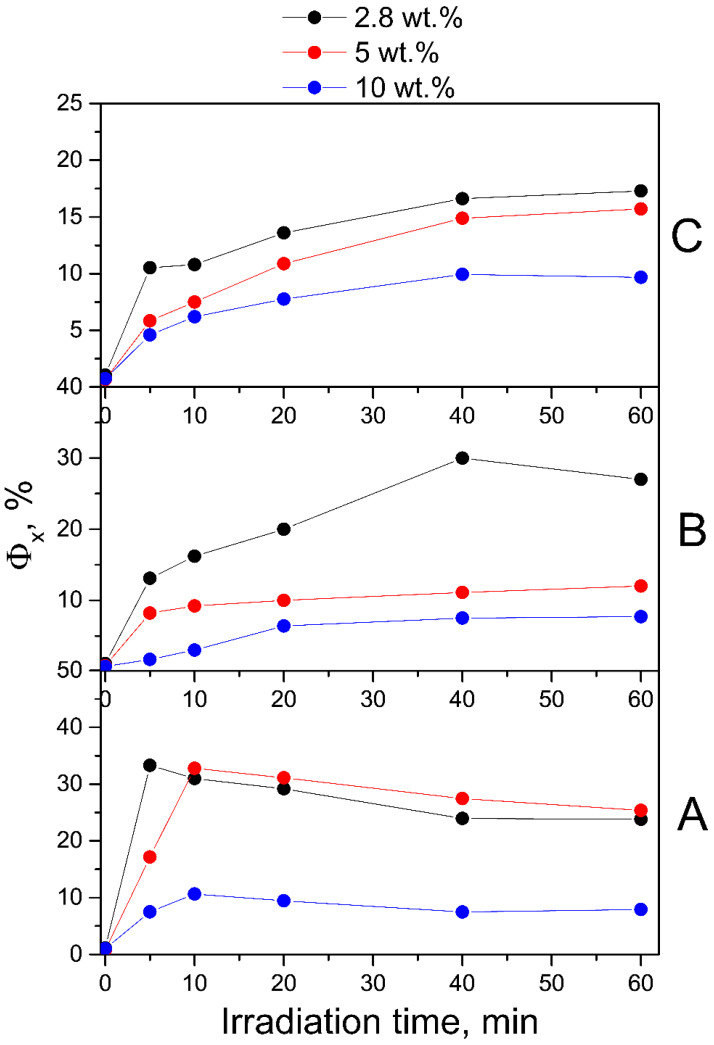
Fluorescence QYs (Φ_x_) of air-equilibrated aqueous solutions of non-irradiated and UV-irradiated FA-PVP-C_60_ conjugates at pH 4.5 (**A**), pH 7.2 (**B**) and pH 10.7 (**C**).

To confirm the observed spectral changes in neutral solutions of irradiated conjugates, we performed fluorescence measurements. The MPT moiety is responsible for the intrinsic light emission of the FA molecule. Notably, the pABGA moiety plays a key role in non-radioactive relaxation via intramolecular charge transfer from photoexcited MPT, thereby quenching the fluorescence emission [58]. The maximum fluorescence emission at 448 nm corresponds to FPT [57,59]. We observed a gradual increase in the fluorescence emission intensity of the irradiated conjugates and then its decrease, without any noticeable changes in the shape of the spectrum (Figure S4, Supplementary Materials). Therefore, the observed increase in the fluorescence of irradiated FA indicates a violation of non-radiative intramolecular excitation transfer, providing additional evidence for the appearance of pterin fragments during the decomposition of FA. The subsequent decrease in fluorescence intensity is associated with the onset of the stage of destruction of the photodegradation products of FA. The obtained fluorescence QY values of FA-PVP-C_60_ conjugates with different fullerene contents at different pH are presented in Figure 5. It can be noted that the QY of fluorescence at neutral pH (Figure 5B) correlates with the obtained proportion of decomposed FA. The decrease in these values helps prevent the oxidation of FPT to CPT, which has a higher fluorescence QY [57]. It should be noted that the QY in all non-irradiated samples was slightly higher than the known value for FA (about 0.5%) [57]. This may be a consequence of the presence of impurities in the FA or its partial degradation during the synthesis of conjugates.

Thus, the photodegradation of FA was confirmed through determination of the fluorescence quantum yield of its photoproducts, which are responsible for the increase in fluorescence intensity under the influence of UV irradiation [27].

#### 2.1.2. Acid Aqueous Solutions

**Figure 6:**
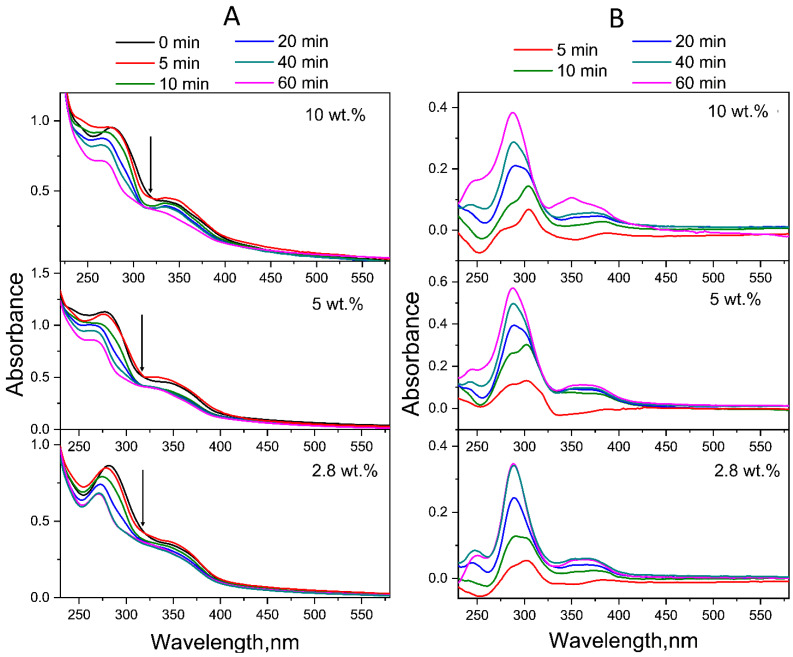
(**A**) Time evolution of the absorption spectra of the FA-PVP-C_60_ conjugates in air-equilibrated aqueous solutions (pH 4.5) under UV irradiation (240–400 nm). Arrows indicate the isosbestic points. (**B**) Experimental difference (ED) spectra obtained by subtracting the spectrum after 5–60 min of photolysis from the FA-PVP-C_60_ conjugates initial spectrum.

The acidic and basic forms of the PT moiety of FA have differences in their electronic structures and charge densities, which can also affect the process of photodegradation of FA at different pH solutions. Figure 6A shows the evolution of the absorption spectra of FA-PVP-C_60_ conjugates exposed to UV irradiation in an acidic medium (pH 4.5). It is possible to note the similarity of spectral changes occurring in acidic and neutral solutions. It is possible to note the similarity of spectral changes occurring during UV irradiation of acidic and neutral solutions of conjugates. Upon UV irradiation of the conjugates in an acidic medium, a gradual decrease in absorption was also observed (Figure 6A), accompanied by a blue shift in the maxima at 282 and 350 nm.

As in the neutral environment, in this case, the changes were more pronounced for the band at 282 nm, responsible for the absorption of FA fragments—both pABGA and PT. The spectrum of FPT in its acidic form had an absorption band centered at 310 nm, and the band centered at approximately 350 nm had a higher relative intensity than that of PT and CPT in both acidic and alkaline media [57]. Moreover, in an acidic environment, the differences in the rate of photodegradation were more pronounced: the ED spectra (Figure 6B) show an increase in the difference between the spectra at 40 and 60 min of irradiation with an increase in the fullerene content in the conjugate. The ED spectra of the conjugate with a fullerene content of 2.8 wt.%, corresponding to 5 and 10 min of irradiation (Figure 6B), contain characteristics of FPT maxima. The ED spectrum of the same conjugate, corresponding to 20 min of irradiation, contains characteristics of CPT maxima. At the same time, on the ED spectra of conjugates with a fullerene content of 5 and 10 wt.%, corresponding to both 20 and 40 min of irradiation and responsible for the absorption of FPT, the shoulder at 310 nm can be noted. This indicates a protective effect of fullerenes on FA under UV irradiation.

The results of the studies, which at the moment according to spectrofluorometry (Figure S5, Supplementary Materials) fluorescence QYs data of all conjugates registered under UV irradiation in an acidic medium, were higher than in a neutral medium (Figure 5). At the same time, the effect of fullerene on the photostability of FA was obvious only at a C_60_ content in the conjugate equal to 10 wt.%. In this case, it is likely that another factor has a decisive influence on the degradation of FA. It is known that in an acidic environment, CPT, both without irradiation and in the absence of oxygen, undergoes decarboxylation with the formation of PT [30], the fluorescence QY of which is higher than that of CPT [57]. The QY of decarboxylation and photooxidation of CPT in an acidic medium were 1.9 · 10^−3^ and 3.2 · 10^−3^, respectively [60].

#### 2.1.3. Alkaline Aqueous Solutions

**Figure 7:**
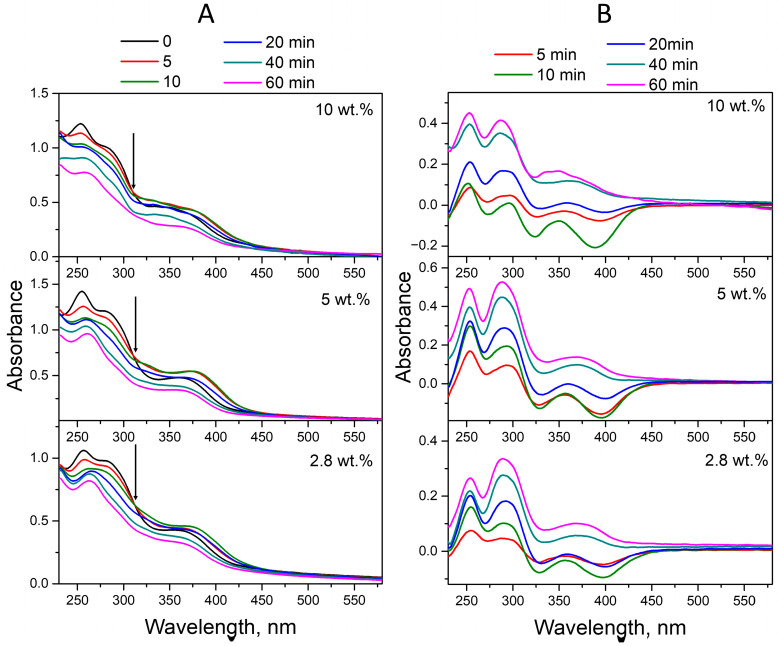
(**A**) Time evolution of the absorption spectra of the FA-PVP-C_60_ conjugates in air-equilibrated aqueous solutions (pH 10.7) under UV irradiation (240–400 nm). Arrows indicate the isosbestic points. (**B**) Experimental difference (ED) spectra obtained by subtracting the spectrum after 5–60 min of photolysis from the FA-PVP-C_60_ conjugates initial spectrum.

Figure 7A shows the absorption spectra of FA-PVP-C_60_ conjugates exposed to UV irradiation in an alkaline medium (pH 10.7). Spectral changes that can be seen during irradiation are different from those observed during photolysis of the conjugate in neutral and acidic media. This is due to the significant difference in the absorption spectra of FPT and CPT in an alkaline medium [57]. No blue shift was observed for the maxima at 256 nm and 283 nm, corresponding to the high-energy bands of PT and pABGA in FA. Instead, a red shift of the PT maximum from 256 to 260 nm was observed, corresponding to the absorption maximum of CPT. The intensity of the peak at 283 nm, mediated by the pABGA fragment in FA, decreased without changing the wavelength. The absorption of the maximum at 365 nm increased in conjugate solutions irradiated for 5–20 min and then decreased along with a blue shift of the maximum to 360 nm. The observed changes correspond to the decomposition of FA with the formation of FPT and pABGA in the first 20 min of photolysis and subsequent oxidation of FPT to CPT. At the same time, a maximum with intense absorption in the 300 nm region can be observed in the ED spectra (Figure 7B), the relative intensity of which increases with increasing irradiation time. These results indicate that under these irradiation conditions, the folate anion does not give the same photoproducts that are found in an acidic medium (FPT, CPT and pABGA), or that these compounds are not the only photoproducts. A similar ED spectrum was previously observed by Thomas et al. during the UV irradiation of an alkaline solution of FA [47]. TLC analysis of irradiated alkaline solutions in addition to FPT and CPT showed the presence of two photostable products, which could not be unambiguously identified. Moreover, the QY of FA photodegradation via the formation of FPC and pABGA and two unidentified products were comparable [47].

The fluorescence QY values of the FA photodegradation products in the conjugates obtained from spectrofluorometry data (Figure S6, Supplementary Materials) in an alkaline medium were lower than in neutral and acidic media (Figure 5), which corresponds to previously obtained data [57].

### 2.2. Radiolysis of FA in FA-PVP-C_60_

One of the most important parameters determining the successful radiation sterilization of a drug is the absorbed dose. The sterilized drug should have a microbial load not exceeding the established values without losing its functionality. In the case of the FA-PVP-C_60_ conjugate, the most important functional characteristics include its ability to target tumor cells overexpressing folate receptors. Therefore, the conjugate should retain the pterin fragment responsible for binding to folate receptors after irradiation. Although the most widely used dose for radiation sterilization is 25 kGy, the lower doses can be used if justified and confirmed according to ISO 11137 [61]. Considering the known data on the radiation resistance of FA, as well as our preliminary experiments, which showed that at doses above 12 kGy FA almost completely degrades. To irradiate conjugates with electrons accelerated to 10 MeV, we chose a dose range from 2 to 8 kGy.

**Figure 8:**
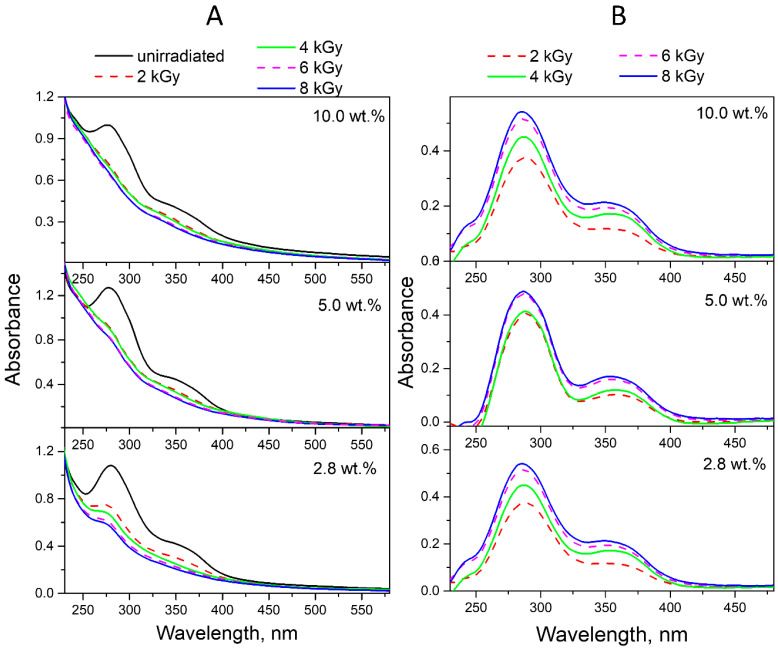
(**A**) Absorption spectra of unirradiated and irradiated (2–8 kGy) FA-PVP-C_60_ conjugates in 7.2 pH buffer. (**B**) Experimental difference (ED) spectra were obtained by subtracting the spectra of conjugates irradiated with a dose of 2–8 kGy from the initial spectrum of FA-PVP-C_60_.

**Figure 9:**
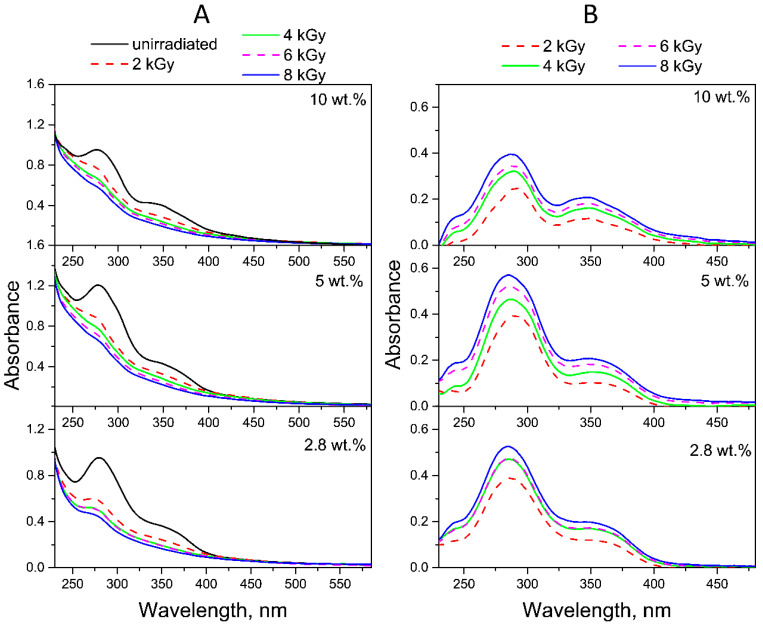
(**A**) Absorption spectra of unirradiated and irradiated (2–8 kGy) FA-PVP-C_60_ conjugates in 4.5 pH buffer. (**B**) Experimental difference (ED) spectra were obtained by subtracting the spectra of conjugates irradiated with a dose of 2–8 kGy from the initial spectrum of FA-PVP-C_60_.

**Figure 10:**
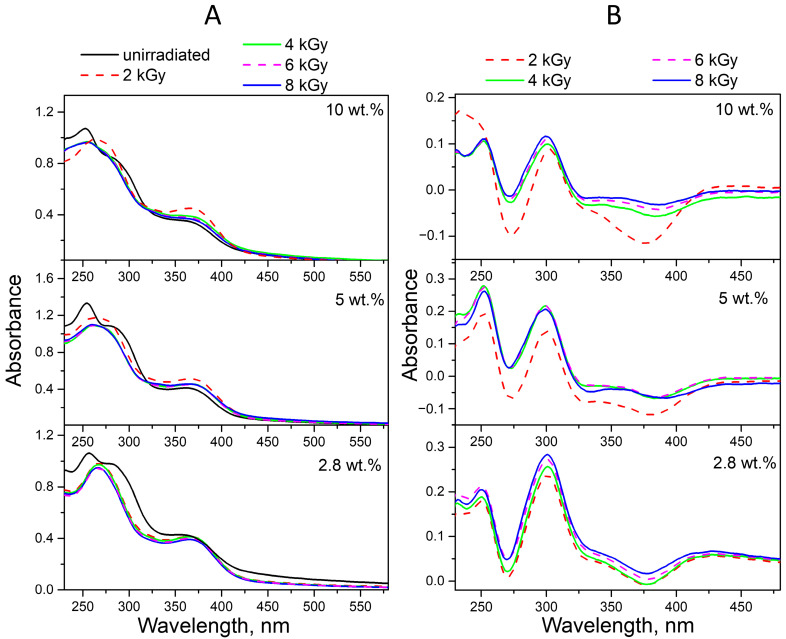
(**A**) Absorption spectra of unirradiated and irradiated (2–8 kGy) FA-PVP-C_60_ conjugates in 10.7 pH buffer. (**B**) Experimental difference (ED) spectra were obtained by subtracting the spectra of conjugates irradiated with a dose of 2–8 kGy from the initial spectrum of FA-PVP-C_60_.

Electron beam irradiation of FA-PVP-C_60_ conjugates at doses from 2 to 8 kGy at different pH promoted more intense FA decomposition when compared to UV irradiation (Figure 8A, Figure 9A and Figure 10A). This phenomenon was expected due to the higher energies and dose rates of electron irradiation compared to UV treatment. It is known that FA is photostable in solutions in the absence of oxygen [30], while electron irradiation of deoxygenated and air-equilibrated solutions resulted in the degradation of FA in both cases [22]. Consequently, during electron irradiation of FA solutions, the formation of highly reactive water radiolysis products—which occurs both in the presence and absence of oxygen—is of decisive importance for the destruction of FA. In radiolyzed FA solutions, no formation of FPT was observed, which was quickly oxidized to CPT by strong oxidizing agents (i.e., hydroxyl radicals) [20,21]. The main contribution to the degradation process of FA by water radiolysis products has been confirmed by the fact that, when irradiating FA in powder form, no noticeable degradation was observed up to a dose of 10 kGy; meanwhile, in solution, FA was completely destroyed at a dose of 5 kGy [22].

The ED spectra obtained by subtracting the spectra of conjugates irradiated with doses of 2–8 kGy in neutral (Figure 8B) and acidic media (Figure 9B) from the spectra of non-irradiated conjugates show that the absorption maxima of the product decreasing during radiolysis are at 290 and 350 nm, which corresponds to the CPT.

In contrast to the FA-PVP-C_60_ conjugates irradiated in acidic and neutral media, the spectra of the conjugates irradiated in an alkaline medium show the absence of maxima characteristic of FA and the presence of maxima at 260 and 360 nm characteristic of CPT (Figure 10A). The ED spectra of the FA-PVP-C_60_ conjugates irradiated in an alkaline medium (Figure 10B) show maxima similar to those observed during photolysis of the FA in the conjugates (Figure 7B). However, it can be noted that in the case of radiolysis, the intensity of the peak at 300 nm increases to a maximum already at a dose of 2 kGy. Taking into account the almost complete absence of the effect of the absorbed dose on the ED spectra of the conjugates irradiated in an alkaline medium, it can be assumed that in this case the products of FA radiolysis are sufficiently radiation-resistant.

**Figure 11:**
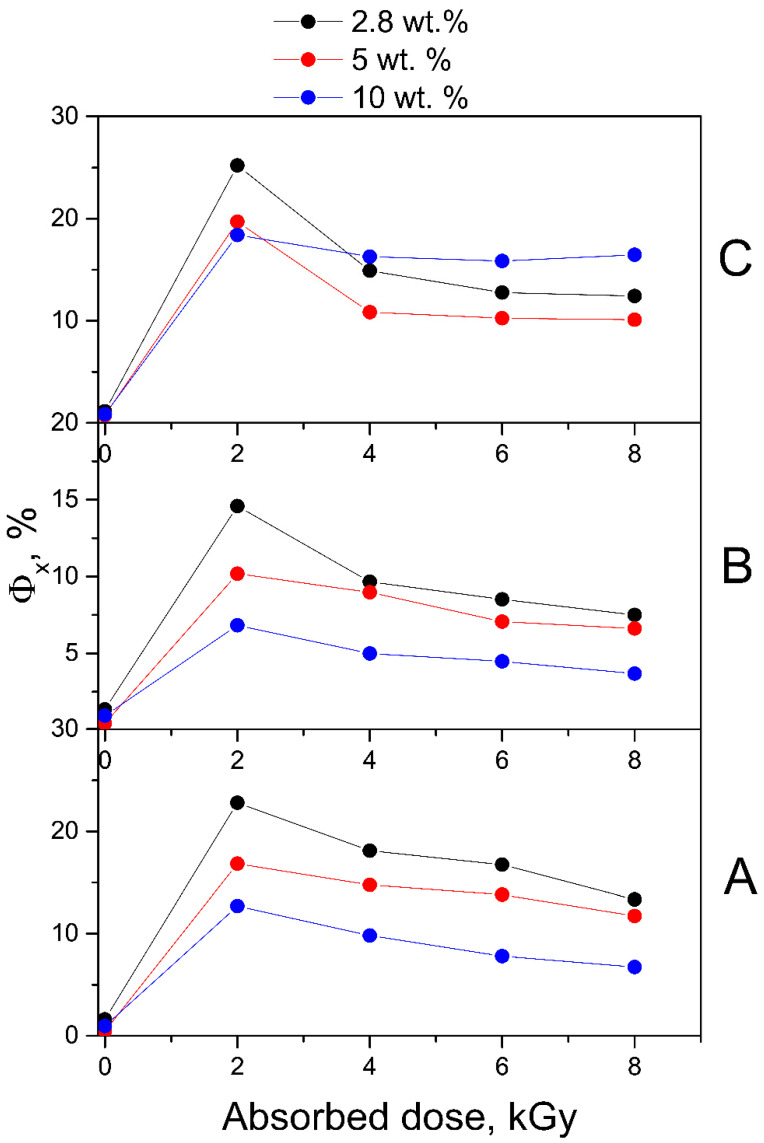
Fluorescence QYs (Φ*_x_*) of air-equilibrated aqueous solutions of non-irradiated and irradiated with doses of 2–8 kGy FA-PVP-C_60_ conjugates at pH 4.5 (**A**), pH 7.2 (**B**) and pH 10.7 (**C**).

In the fluorescence spectra of the FA-PVP-C_60_ conjugates (Figures S7–S9, Supplementary Materials), a slight increase in fluorescence intensity was observed upon irradiation with a dose of 2 kGy. Then, as the absorbed dose increased, a decrease in fluorescence intensity occurred, indicating the destruction of fluorescent degradation products of FA. We observed a blue shift in the fluorescence emission maximum in all spectra (Figure S7–S9, Supplementary Materials) of irradiated samples, which would indicate the formation of CPT [19]. Thus, the protective effect of fullerene on FA during irradiation was more clearly manifested in the spectrofluorometry data: as can be seen in Figure 11, the fluorescence of QYs radiolysis products decreases with an increase in the amount of fullerene in the conjugate.

The concentrations of destroyed FA were calculated using the molar extinction coefficients corresponding to each conjugate, as well as the difference of the absorption values at the maxima at 280 nm and 350 nm between unirradiated and irradiated samples at a dose of 2 kGy. As a quantitative assessment of the effect of fullerene on the radiolysis of FA in the FA-PVP-C_60_ conjugate, the radiation chemical yield of FA destruction was determined according to the following Equation (1):

(1) $$G\left(-FA\right)=9.65\times 10^{6}\frac{c\left(-FA\right)}{D\times \rho },$$

where *D* is the absorbed dose, Gy; ρ is the solution density; and *c* is the concentration of destroyed FA (mol/L).

The obtained radiation chemical yield of FA destruction values at different pH, averaged over the two wavelengths (280 and 350 nm), are given in Table 1. The radiation chemical yields of FA destruction in an alkaline medium were not calculated, since both the spectra of the irradiated conjugates and the ED spectra did not correspond to the FA spectrum; therefore, the concentration of the decreasing FA could not be adequately determined.

As can be seen from Table 1, with an increase in the amount of fullerene in the FA-PVP-C_60_ conjugates, the radiation chemical yield of FA destruction decreases slightly. It is known from the literature that the radiation chemical yield of destruction of native FA in water at pH 7.4, irradiated in various atmospheres, ranges from 0.77 to 3.38 molecules/100 eV [62]. These data indicate that in the presence of fullerene, which is known for its antioxidant and antiradical properties, FA is more resistant to the effects of reactive oxygen species formed in aqueous solutions under the influence of ionizing radiation.

**Figure 12:**
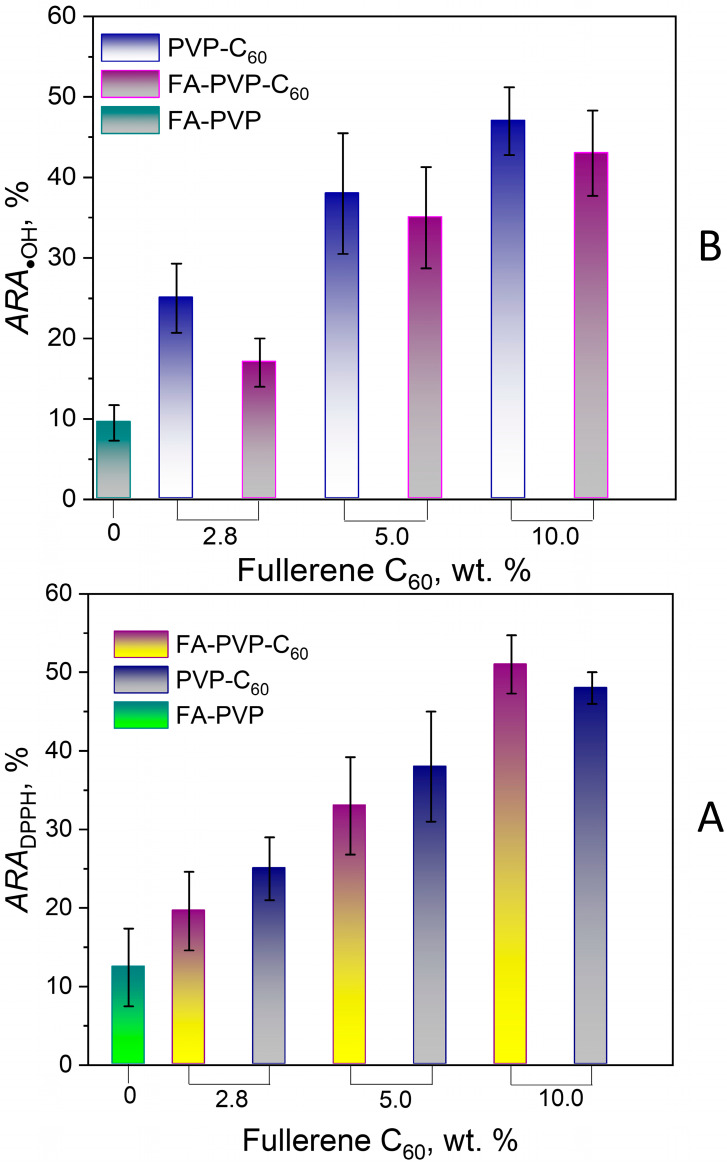
Radical scavenging activity of FA-PVP-C_60_ and intermediate conjugates FA-PVP and PVP-C_60_. (**A**) DPPH radical scavenging activity; (**B**) hydroxyl radical scavenging activity. The data are presented as mean values ± SD (*n* = 3 independent experiments).

To confirm this conclusion, we assessed the ability of the FA-PVP-C_60_ and intermediate FA-PVP and C_60_-PVP conjugates to scavenge the model free radical DPPH, as well as the hydroxyl radical. Figure 12 shows the dependence of the conjugate’s radical scavenging ability on the fullerene content in the conjugate. The antiradical activity (ARA) of the conjugates increased with increasing fullerene content. The ARA values of the PVP-C_60_ intermediate conjugates turned out to be comparable with that of the FA-PVP-C_60_ conjugates (Figure 12A,B). Despite the fact that FA is known for its antioxidant properties [63], the intermediate conjugate FA-PVP demonstrated only moderate ARA against DPPH (Figure 12A) and hydroxyl radicals (Figure 12B). At the same time, we did not observe the ARA of the conjugate FA-PVP-C_60_ equal to the sum of the intermediate conjugates ARA. Probably, the ARA of the conjugates also depends on the accessibility of the reaction centers involved in the scavenging of free radicals. Hydrophobic interactions of poorly soluble components of the conjugate (fullerene and FA) can lead to less accessibility of these molecules for free radicals.

These data, together with the obtained radiation chemical yield of FA destruction values, indicate that during the radiolysis of FA in FA-PVP-C_60_, the observed protective mechanism of action is associated with the ability of fullerene C_60_ to scavenge hydroxyl radicals.

## 3. Materials and Methods

### 3.1. Materials

FA (pure 98 wt.%), 2-diphenyl-1-picryl-hydrazyl (DPPH) and PVP K30 (with a molecular weight of 40.0 kDa, according to the manufacturer’s information) were provided by Sisco (Mumbai, India). Quinine hemisulfate salt monohydrate was purchased from Sigma Aldrich (St. Louis, MO, USA). Sulfuric acid, iron (II) sulfate 7-hydrate and hydrogen peroxide were obtained from Vecton (St. Petersburg, Russia). Methyl violet was provided by LenReactiv (St. Petersburg, Russia). The synthesis of the FA-PVP-C_60_ conjugates has been described previously [14]. To assess the effect of fullerene C_60_ on the degradation of FA and FA-PVP-C_60_ conjugates with different fullerene contents (i.e., 2.8, 5 and 10 wt.%), they were prepared. The FA content in all conjugates was 8.9 wt.%.

### 3.2. Methods

#### 3.2.1. Characterization of FA-PVP-C_60_

The crystal structure of the samples was confirmed using powder X-ray diffractometry on a DRON-8 diffractometer (Bourevestnik JSC, St. Petersburg, Russia). To record the FTIR spectra, an IRTracer-100 infrared Fourier spectrometer (Shimadzu, Kyoto, Japan) with an ATR attachment (Shimadzu, Kyoto, Japan) was used. The spectra were recorded at 32 scans per spectrum and a resolution of 4 cm^−1^ in the range 4000–400 cm^−1^.

#### 3.2.2. UV Irradiation of FA-PVP-C_60_

To study the photolysis of FA, aqueous solutions of FA-PVP-C_60_ conjugates with different contents of fullerene C_60_ (from 2.8 to 10 wt.%) with a concentration of 200 μg/mL were irradiated simultaneously with two lamps: OUFK-320/400-03 (effective radiation range: 300–400 nm, maximum 350 nm, power 0.7 mW/cm^2^) and OUFK-09-1 (effective radiation range: 205–315 nm, maximum 254 nm, power 1.1 mW/cm^2^). For irradiation, conjugate solutions were prepared in acidic (pH 4.5, 0.05 M potassium dihydrogen phosphate solution), neutral (pH 7.2, 0.01 M phosphate-buffered saline), and alkaline (pH 10.7, 0.2 M sodium carbonate solution) media. The solutions were irradiated in 10 mm quartz cuvettes (4 mL) in a closed box at a distance of 3 cm from the lamps for 0–60 min. All experiments were carried out under constant dim lighting to avoid uncontrolled external exposure to light.

#### 3.2.3. E-Beam Irradiation of FA-PVP-C_60_

To study the radiolysis of FA, the air-equilibrated aqueous solutions at different pH (4.5, 7.2, and 10.7) of FA-PVP-C_60_ with a concentration of 200 μg/mL were irradiated in dark glass vials at doses of 2, 4, 6, and 8 kGy. Irradiation was performed using 10 MeV accelerated electrons obtained with a Mevex MB10-30SC900 linear accelerator (STERIS AST Equipment and Technologies, Mentor, OH, USA). Dose control was carried out using a film dosimeter CO(PDE) 1-10 (VNIIFTRI, Zelenograd, Russia).

#### 3.2.4. Absorption and Fluorescence Spectroscopy

The degradation of FA was determined according to changes in the absorbance spectra of FA solutions obtained using a UV–Vis SF-2000 spectrophotometer (OKB Spectr LLC, St. Petersburg, Russia). To obtain fluorescence emission spectra in the range of 400–600 nm, a CM2203 spectrofluorometer (Solar CJSC, Minsk, Republic of Belarus) was used at an excitation wavelength of 385 nm and 5 nm excitation and emission slits.

#### 3.2.5. Fluorescence Quantum Yield Calculation

The calculation of the fluorescence QY allows us to estimate the efficiency of the emission. The QY of FA and its photo- and radioproducts was calculated using the relative method, with quinine sulfate in 0.05 M H_2_SO_4_ as a reference fluorophore [64]. The samples and reference were measured under the same experimental conditions: absorption at identical excitation wavelength (λ_ex_ = 380 nm), and excitation and emission monochromator slits. The QY (%) was calculated using the following Equation (2) [64]:

(2) $$\Phi_{x}=\Phi_{ref}\cdot (\frac{A_{ref}\cdot I_{x}}{A_{x}\cdot I_{ref}})\cdot \frac{n_{x}^{2}}{n_{ref}^{2}},$$

where Φ_ref_ stands for the reference QY (54% for quinine sulfate [65]), and *A*_ref_ and *A*_x_ stand for absorption at the excitation wavelengths of the reference and FA-PVP-C_60_ conjugate, respectively; *I*_x_ and *I*_ref_ stand for the fluorescence peak areas of the sample and the reference, respectively; and *n* stands for the refraction index of the solvent used to dissolve the sample and the reference. To avoid the internal filter effect, all solutions were diluted so that the absorption at the fluorescence excitation wavelength did not exceed 0.1.

#### 3.2.6. DPPH Scavenging Activity

The antiradical activity of FA-PVP-C_60_, FA-PVP, and PVP-C_60_ with respect to the stable radical DPPH was studied using UV–Vis spectrometry on an SF-2000 spectrophotometer (OKB Spectr LLC, Saint-Petersburg, Russia). A detailed description of the experiment has been presented previously [14]. The antiradical activity of conjugates *ARA*_DPPH·_ was also calculated at 30 min after the start of the ^•^DPPH reduction reaction using Equation (3):

(3) $${ARA}_{DPPH\cdot }=\frac{\left(A_{DPPH\cdot }-A_{sample}\right)}{A_{DPPH\cdot }}\cdot 100\%,$$

where $A_{sample}$ and $A_{DPPH\cdot }$ are the absorbances of 130 μM DPPH solutions in the presence and absence of 1000 μg/mL FA-PVP-C_60_ (FA-PVP, PVP-C_60_), respectively. The experiments were independently repeated three times.

#### 3.2.7. Hydroxyl Radical Scavenging Activity

The ability of FA-PVP-C_60_, FA-PVP, and PVP-C_60_ to scavenge hydroxyl radicals was studied using spectrophotometric determination of the destruction degree of methyl violet (MV) when it reacted with ^•^OH [66]. The ^•^OH was generated during the Fe^2+^/Fe^3+^, Fenton reaction occurring in the presence of H_2_O_2_. A mixture of equal volumes of aqueous solutions of 30 μM MV, 50 mM H_2_O_2_ and 0.4 mM iron (II) sulfate 7-hydrate in deionized water, as well as an aqueous solution of FA-PVP-C_60_ at a concentration of 1000 μg/mL, was stirred for 30 min. A similar mixture was used as a blank experiment, replacing the MV solution with deionized water. The absorbance of the samples was measured at 585 nm using an SF-2000 spectrophotometer (OKB Spectr LLC, St. Petersburg, Russia). The *A*_•OH_ absorbance was measured by replacing the FA-PVP-C_60_ solution with an equal volume of deionized water. The hydroxyl radical scavenging activity (%) *ARA*_•OH_ was calculated using Equation (4):

(4) $${ARA}_{•OH}=\frac{\left(A_{sample-A_{•OH}}\right)}{A_{sample}}\cdot 100\%,$$

where $A_{sample}$ and $A_{•OH}$ are absorption at 585 nm at 30 min after the start of the reaction in the presence and absence of FA-PVP-C_60_ (FA-PVP, PVP-C_60_), respectively. The experiments were repeated independently three times.

## 4. Conclusions

FA, which is used as a targeting ligand in many antitumor drugs, exhibits instability under the influence of radiation, making it necessary to observe special measures during the synthesis, storage, and usage of drugs based on it. Our research revealed that fullerene C_60_ has an inhibitory effect on the process of FA photolysis in the FA-PVP-C_60_ conjugate, which was confirmed by UV–Vis and spectrofluorometry data. Under electron irradiation of the conjugate, the effect of fullerene on FA radiolysis was also revealed. It was found that the radiation chemical yield of FA destruction decreases with an increase in fullerene content in the FA-PVP-C_60_ conjugate, and the obtained value was significantly lower than that for native FA. Our study confirmed that the bonds between components of FA, such as PT and pABGA, are the most sensitive to irradiation, indicating the most probable products of photolysis are pABGA and FPT, the latter of which was oxidized to CPT, which is also characteristic of native FA. At the same time, the study conducted in this work has a limitation: for the quantitative assessment of the destruction of FA and the formation of photolysis products, we were unable to conduct HPLC. This turned out to be problematic due to the complexity of separating the photolysis products in the conjugates due to the presence of the polymer in it. Our further efforts will be aimed at solving this problem.

In the process of electron irradiation, active oxygen species that are formed during the radiolysis of water significantly contribute to the destruction of FA. Fullerene, which exhibits strong antioxidant properties, suppressed the degradation of FA during photolysis and radiolysis by accepting the free radicals generated as a result of these processes. The obtained results indicate that the effective radiation sterilization of conjugates containing FA requires approaches that help to reduce the radiation yield of FA destruction. As it is well known that the fragment responsible for the absorption of FA by folate receptors on cells is PT, it is important that the radiation-sterilized FA retain this property to maintain its efficacy as a targeting vector for drug delivery.

## Figures and Tables

**Table 1:** Effect of the content of fullerene C_60_ in the composition of the FA-PVP-C_60_ conjugate and pH on the radiation chemical yield of FA destruction.

pH	*G*(−FA) × 10^2^, Molecules/100 eV		
	Fullerene Content in the Conjugate, wt.%		
	2.8	5.0	10.0
4.5	7.0 ± 0.1	5.0 ± 0.1	4.0 ± 0.2
7.2	6.0 ± 0.1	5.8 ± 0.1	5.5 ± 0.1
10.7	not defined		

## Data Availability

Data are contained within the article.

## Supplementary Materials

The following supporting information can be downloaded at https://www.mdpi.com/article/10.3390/molecules30132718/s1, Figure S1: UV-Vis spectra of PVP-C_60_ conjugates in DMF. Figure S2: FTIR-spectra of FA-PVP-C_60_ conjugates with different fullerene content: 2.8 wt.% (A), 5.0 wt.% (B), and 10.0 wt.% (C). Figure S3: XRD-spectra of FA-PVP-C_60_ conjugates with different fullerene content and pure PVP. Figure S4: Fluorescence emission spectra of UV-irradiated FA-PVP-C_60_ conjugates at 7.2 pH with fullerene contents of 2.8 wt.% (A); 5 wt.% (B); and 10 wt.% (C). Figure S5: Fluorescence emission spectra of UV-irradiated FA-PVP-C_60_ conjugates at 4.5 pH with fullerene contents of 2.8 wt.% (A); 5 wt.% (B); and 10 wt.% (C). Figure S6: Fluorescence emission spectra of UV-irradiated FA-PVP-C_60_ conjugates at 10.7 pH with fullerene contents of 2.8 wt.% (A); 5 wt.% (B); and 10 wt.% (C). Figure S7: Fluorescence emission spectra of E-beam-irradiated FA-PVP-C_60_ conjugates at 7.2 pH with fullerene contents of 2.8 wt.% (A); 5 wt.% (B); and 10 wt.% (C). Figure S8:Fluorescence emission spectra of E-beam-irradiated FA-PVP-C_60_ conjugates at 4.5 pH with fullerene contents of 2.8 wt.% (A); 5 wt.% (B); and 10 wt.% (C). Figure S9. Fluorescence emission spectra of E-beam-irradiated FA-PVP-C_60_ conjugates at 10.7 pH with fullerene contents of 2.8 wt.% (A); 5 wt.% (B); and 10 wt.% (C).
