# Supplementary material for: Degradation of Folic Acid in the Composition of a Conjugate with Polyvinylpyrrolidone and Fullerene C60 Under UV and E-Beam Irradiation
Source: Molecules. 2025 Jun 24;30(13):2718. doi: 10.3390/molecules30132718 (PMC12251273; doi:10.3390/molecules30132718)
Supplement: Supplementary file 1 [file molecules-30-02718-s001.zip › molecules-3656387-supplementary.pdf]

Supplementary Materials

Article

Degradation of folic acid in the composition of a conjugate with polyvinylpyrrolidone and fullerene C_{60} under UV- and E-beam irradiation

Alina A. Borisenkova [1,2]*, Dmitriy V. Baykov [1], Anna V. Titova [1,2], Vadim V. Bakhmetyev [3], Maria A. Markova [1,2], Zhanna B. Lyutova [1,2], Anton V. Popugaev [1,2], Vladislav S. Khaleev [4] and Victor P. Sedov [2]

[1]Radiation Technology Department, St. Petersburg State Institute of Technology (Technical University); 190013, St. Petersburg, Russia
[2]Petersburg Nuclear Physics Institute named by B.P. Konstantinov of National Research Centre “Kurchatov Institute”; 188300, Gatchina, Russia
[3]Department of Theory of Materials Science, St. Petersburg State Institute of Technology (Technical University); 190013, St. Petersburg, Russia
[4]Infochemistry Scientific Center, ITMO University; 191002, St. Petersburg, Russia
*Correspondence: to whom correspondence should be addressed.

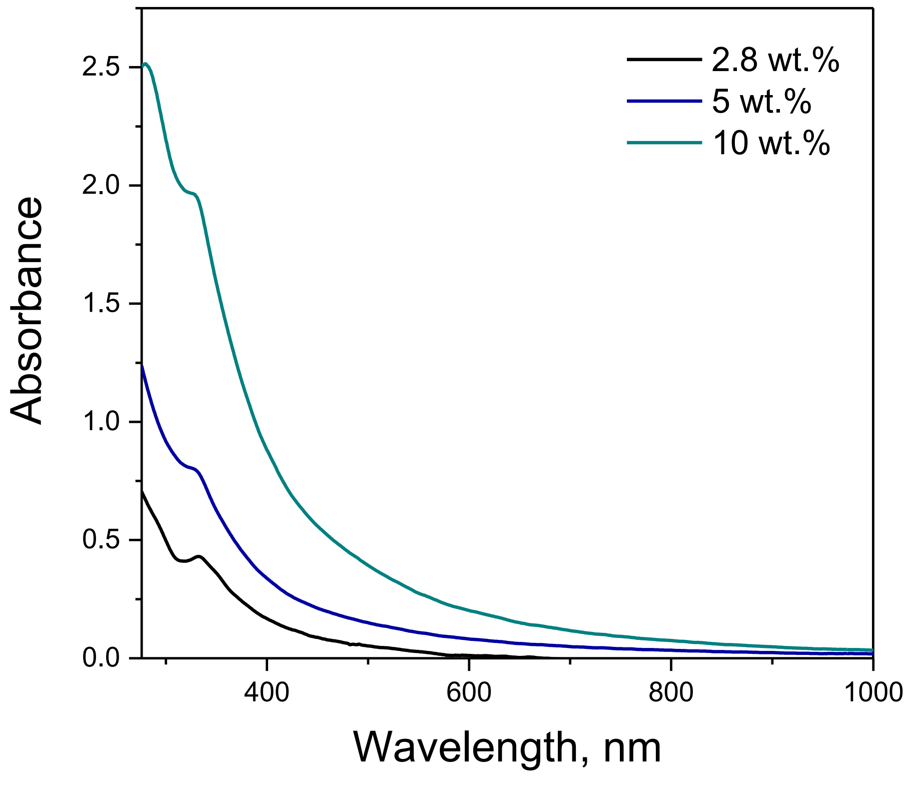

Figure S1. UV-Vis- spectra of PVP-C_{60} conjugates in DMF

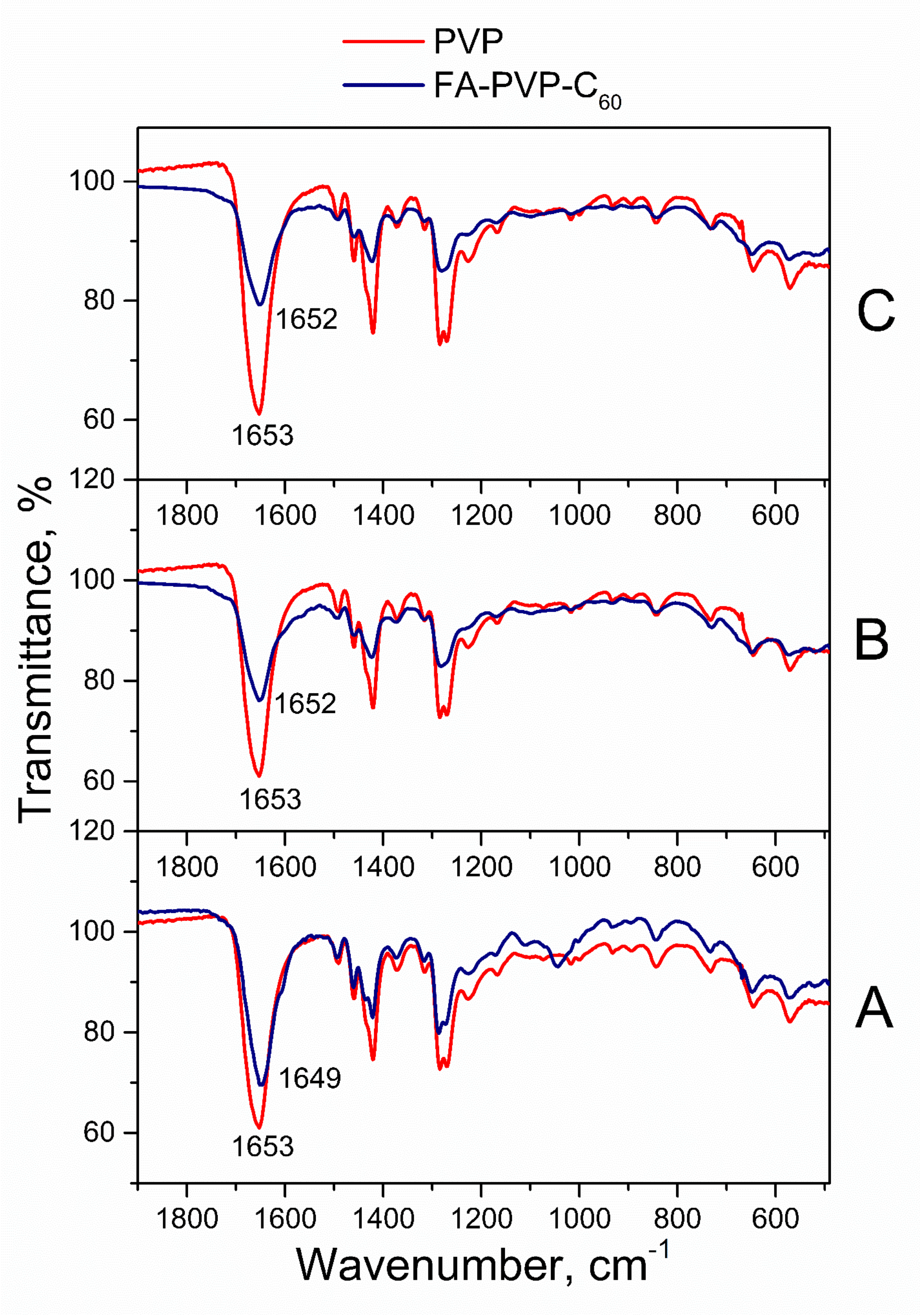

Figure S2. FTIR-spectra of FA-PVP-C_{60} conjugates with different fullerene content: 2.8 wt.% (A), 5.0 wt.% (B), and 10.0 wt.% (C).

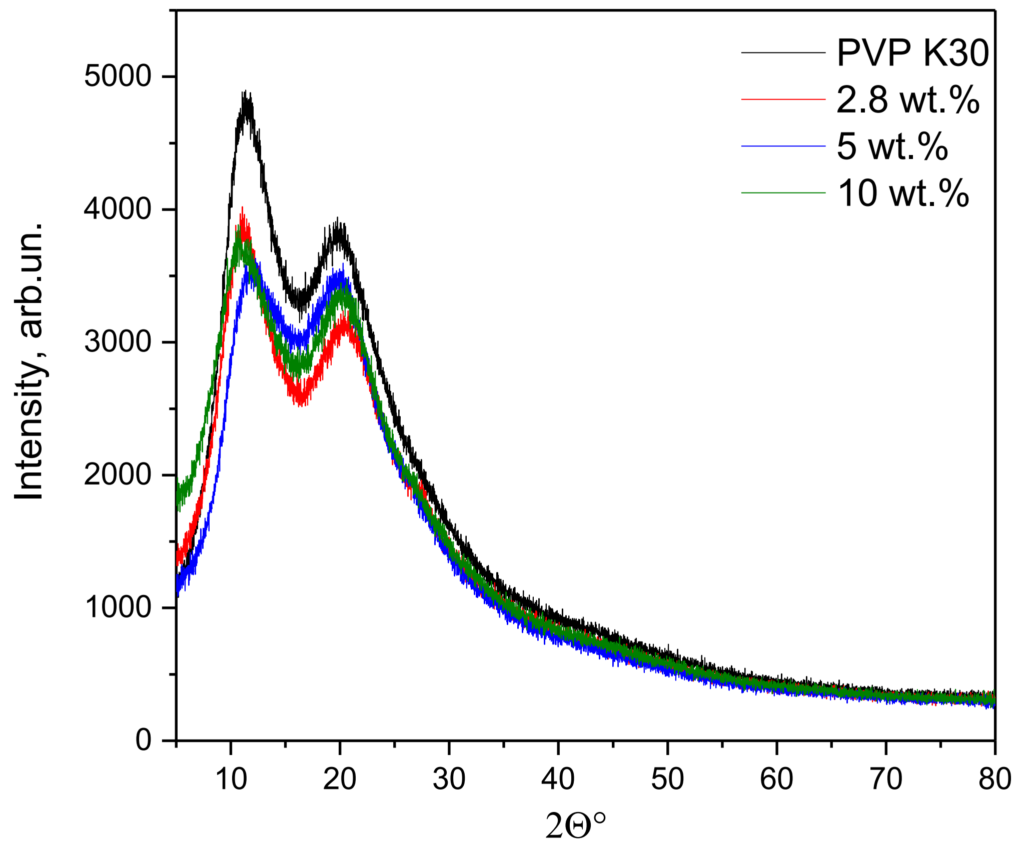

Figure S3. XRD-spectra of FA-PVP-C_{60} conjugates with different fullerene content and pure PVP

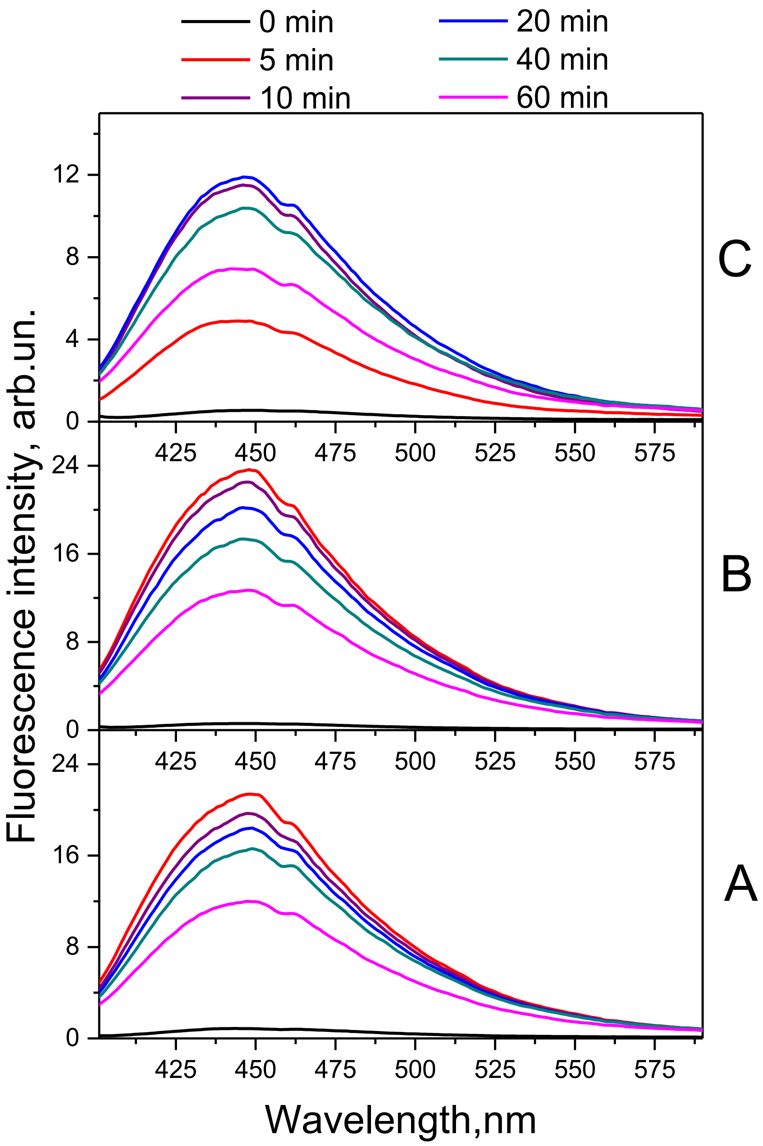

Figure S4. Fluorescence emission spectra of UV-irradiated FA-PVP-C_{60} conjugates at 7.2 pH with fullerene contents of 2.8 wt.% (A); 5 wt.% (B); and 10 wt.% (C).

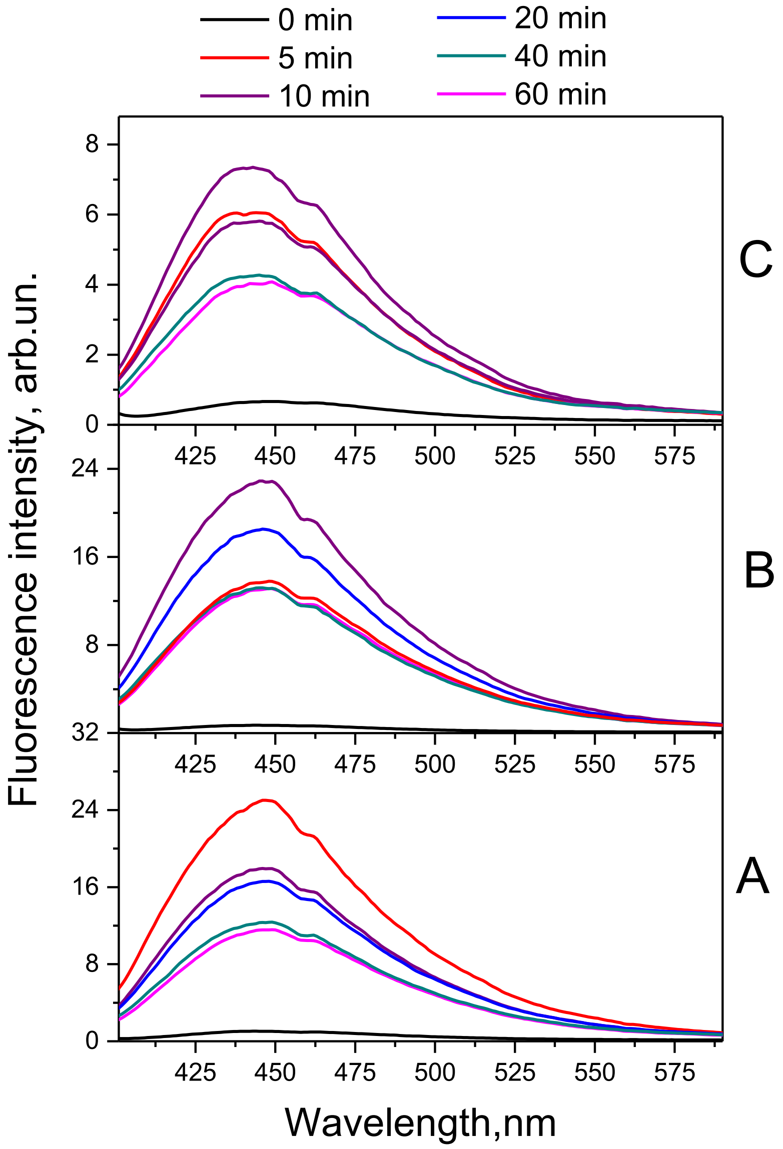

Figure S5. Fluorescence emission spectra of UV-irradiated FA-PVP-C_{60} conjugates at 4.5 pH with fullerene contents of 2.8 wt.% (A); 5 wt.% (B); and 10 wt.% (C).

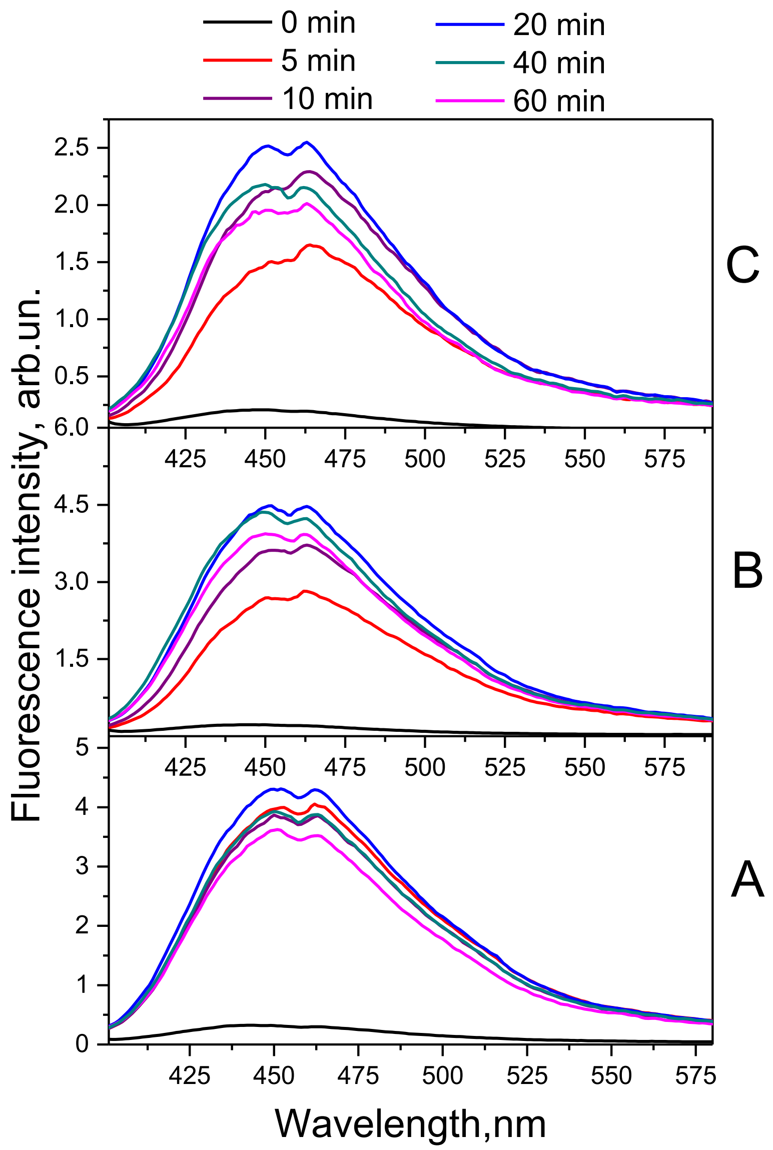

Figure S6. Fluorescence emission spectra of UV-irradiated FA-PVP-C_{60} conjugates at 10.7 pH with fullerene contents of 2.8 wt.% (A); 5 wt.% (B); and 10 wt.% (C).

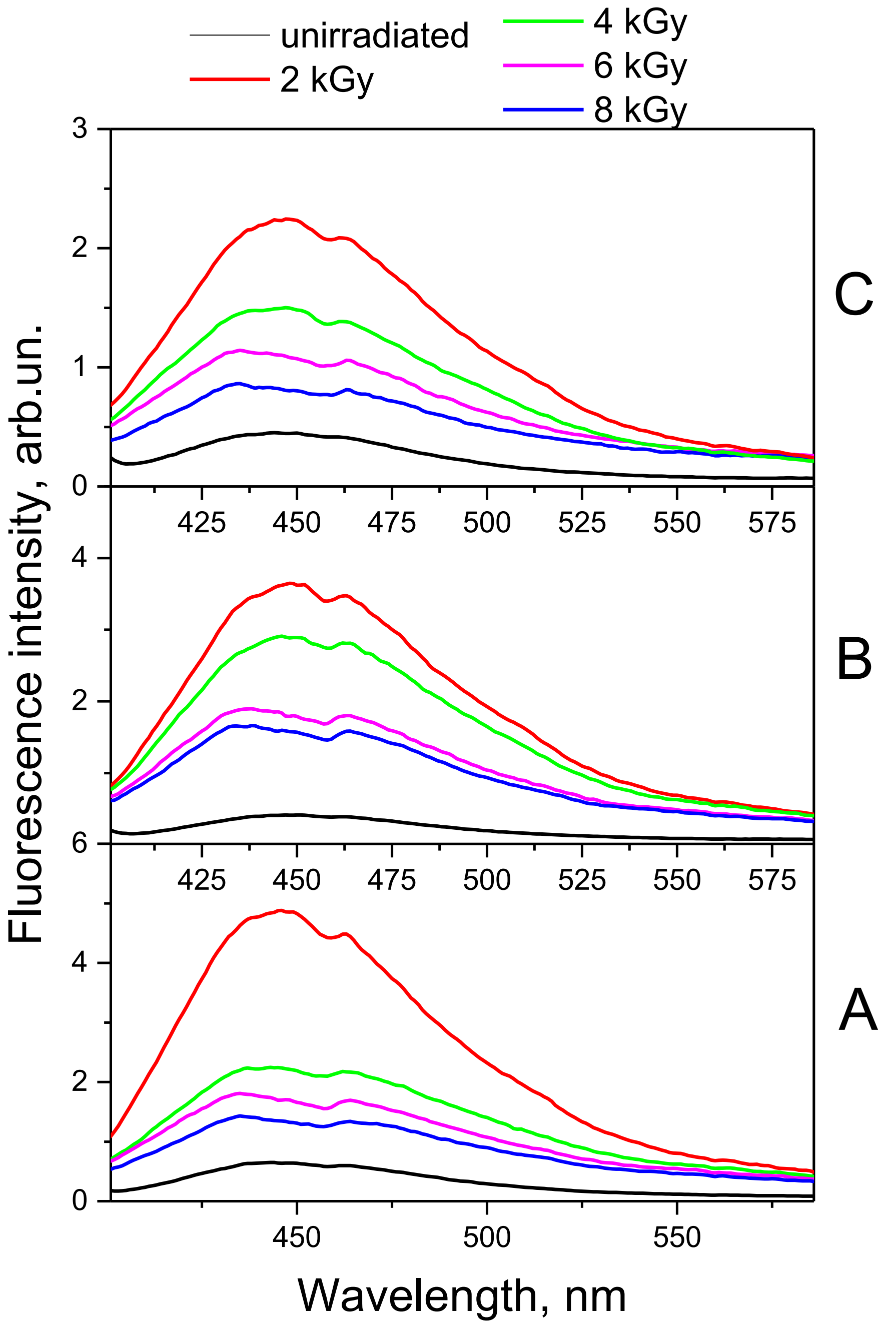

Figure S7. Fluorescence emission spectra of E-beam-irradiated FA-PVP-C_{60} conjugates at 7.2 pH with fullerene contents of 2.8 wt.% (A); 5 wt.% (B); and 10 wt.% (C).

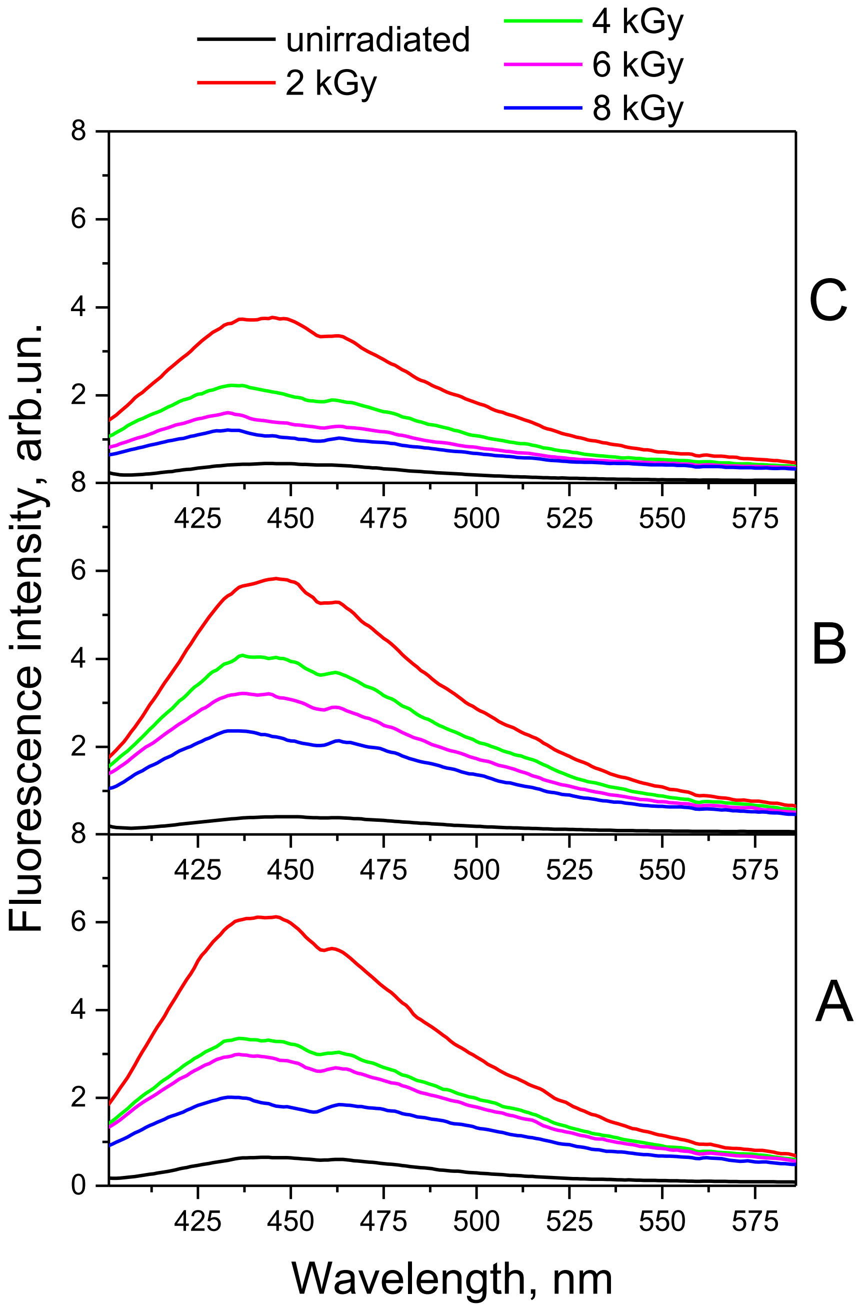

Figure S8. Fluorescence emission spectra of E-beam-irradiated FA-PVP-C_{60} conjugates at 4.5 pH with fullerene contents of 2.8 wt.% (A); 5 wt.% (B); and 10 wt.% (C).

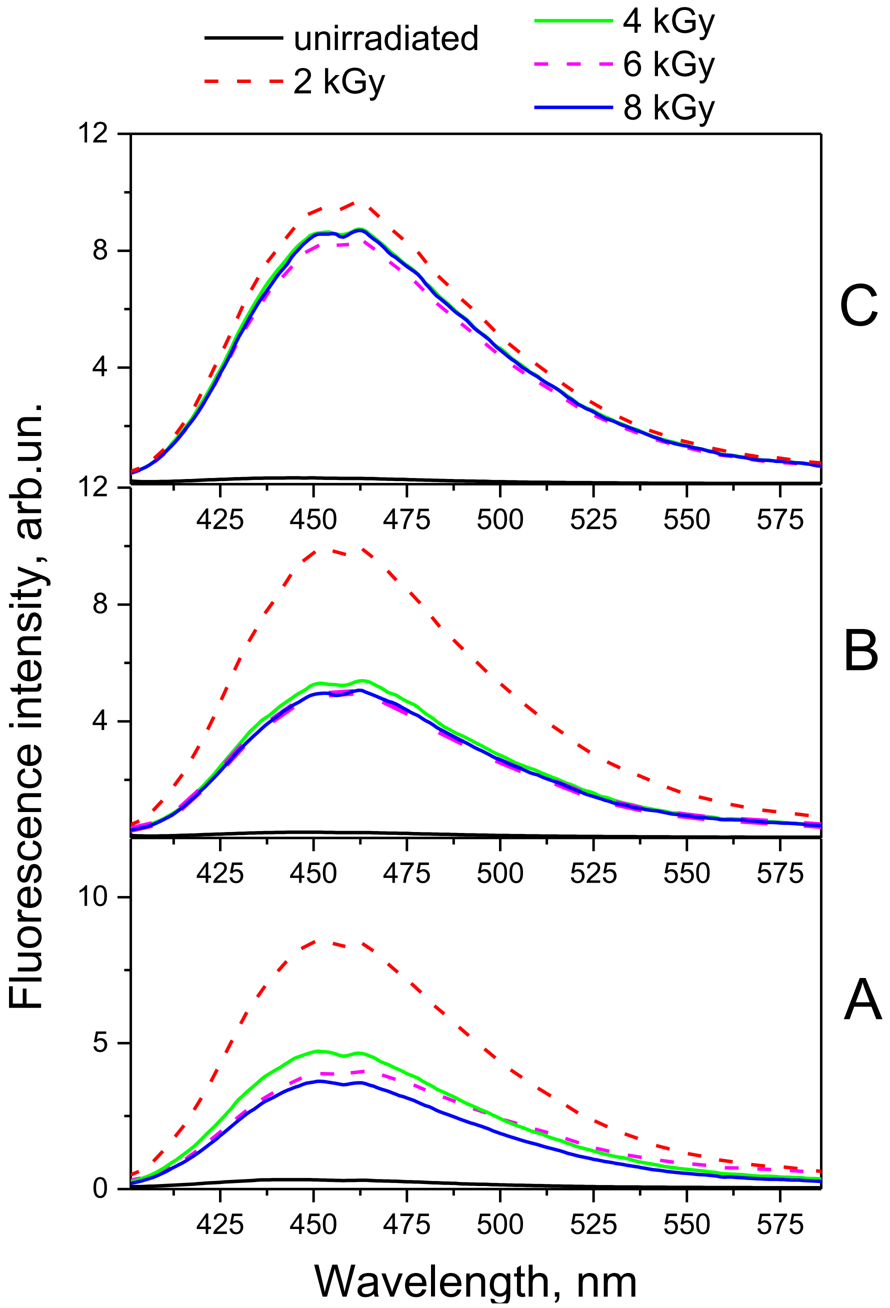

Figure S9. Fluorescence emission spectra of E-beam-irradiated FA-PVP-C_{60} conjugates at 10.7 pH with fullerene contents of 2.8 wt.% (A); 5 wt.% (B); and 10 wt.% (C).
